# New Horizon of Intervention in Congenital Heart Disease: AFR in a Complex Cyanotic Patient

**DOI:** 10.1155/2020/8897101

**Published:** 2020-12-23

**Authors:** Zahra Khajali, Homa Ghaderian, Ata Firoozi, Zahra Hosseini, Maryam Aliramezany

**Affiliations:** ^1^Rajaie Cardiovascular Medical and Research Center, Iran University of Medical Sciences, Tehran, Iran; ^2^Cardiovascular Research Center, Institute of Basic and Clinical Physiology Sciences, Kerman University of Medical Sciences Kerman, Iran

## Abstract

Double-inlet left ventricle (DILV) is the commonest form of the anatomic univentricular heart which has different ventriculoarterial connection; generally, the most prevalent type is DILV with the hypoplastic right ventricle on the left side. The disease is associated with several heart defects, and the treatment method is different based on the anatomy of the heart, but treatment methods are almost always palliative. In this paper, we described one adult patient with DILV and severe left AV (atrioventricular valve) stenosis who is managed with a novel palliative intervention; it means AFR (atrial flow regulator) device implantation for the first time.

## 1. Introduction

DILV is the most common form of single ventricle which both AV (atrioventricular) valves communicate into a common chamber; the prevalence of this rare anatomical anomaly is reported about 1.5-5%. There are three primary subtypes of DILV based on the relationship of the great arteries:

Type I: DILV has normally related great arteries

Type II: has a rightward and anterior aorta and rightward outlet chamber

Type III: has a leftward anterior aorta

DILV with a hypoplastic subpulmonary, rightward RV and normally related great arteries (type I) is classically referred to as the “Holmes heart” and is relatively rare. In type II, the outlet chamber is anterior and rightward, consistent with D-looped ventricles, and there is D-transposition of the great arteries (SDD). This type is associated with the obstruction of the bulboventricular foramen and, therefore, subaortic stenosis, because the aorta arises from the small chamber. Arch anomalies are reported in approximately 50% of cases.

Type III is the most common form of DILV, which consists of DILV with a left-sided, subaortic, hypoplastic right ventricle (L-loop ventricles) and L-transposition of the great arteries (SLL). Subaortic stenosis is present in approximately 67% of patients with this morphology due to a small bulboventricular foramen or obstruction by the left AV valve tissue [1–3].

Associated cardiac anomalies are commonly seen in this complex anatomical disease, such as aortic coarctation or interruption in which the systemic outflow traverses from the bulboventricular foramen, which has a tendency to narrow over time, double-outlet arterial connections, pulmonary atresia, valvular and subvalvular pulmonary stenosis, subaortic obstruction in V-A discordant type, left or right A-V valve stenosis, and conduction abnormalities [4].

Herein, we aimed to report a patient with a functional univentricle who was diagnosed with DILV type III association with severe left A-V valve stenosis who is the first Iranian experience of AFR implantation for congenital heart disease patients which led to improve in congestion symptoms and oxygenation.

## 2. Case Report

A 38-year-old man who was the known case of DILV and Eisenmenger syndrome and was managed with optimal medical therapy referred with progressive dyspnea (NYHA functional class IV) and cyanosis to our clinic for better evaluation. In physical exam, his hemodynamic was stable with So2: 70%. Obviously, he had severe clubbing and central cyanosis; in cardiac exam, left lateral deviation of the LV apex with loud P2 at the base of the heart and diastolic murmur grade II was heard in the left sternal border and apex.

**Figure 1:**
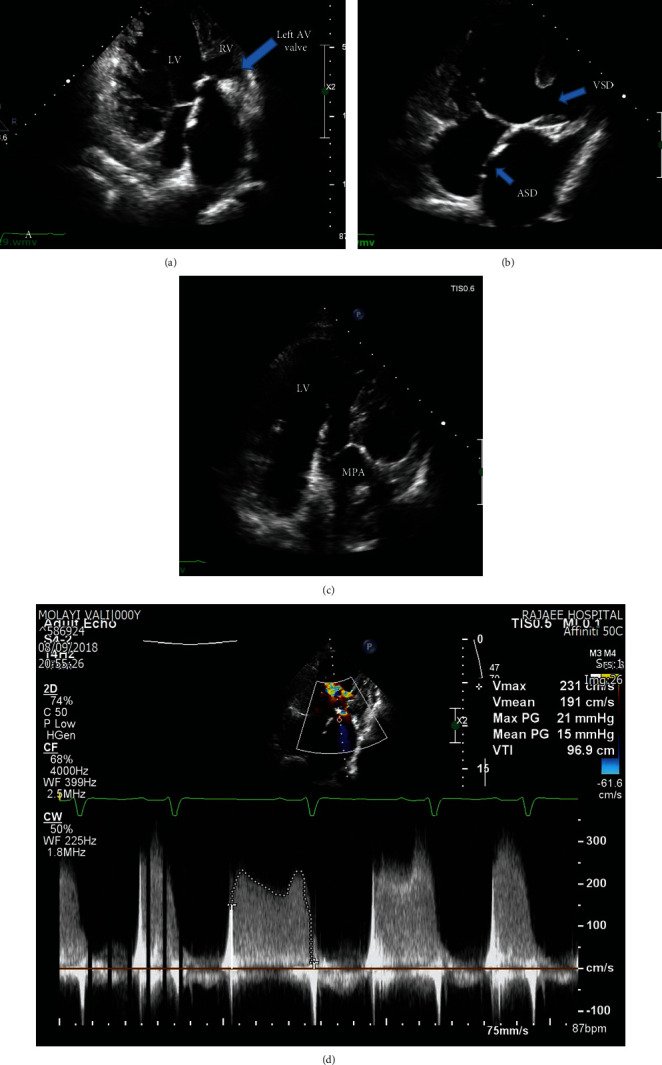
Transthoracic echocardiography in 4-chamber view shows (a) morphologic LV which is the right side of rudimentary RV, (b) large inlet type VSD, very small ASD, (C) PA originate from the main ventricle with confluent PABs, (d) Thick and hypo plastic left AV valve severe stenosis (MG:15, PG:21).

Transthoracic echocardiography demonstrated abdominal and atrial situs solitus, levocardia, L-looped, A-V, and V-A discordance. Morphologic LV (main ventricle) was the right side of the rudimentary right ventricle with a ejection fraction of ~35%. A large inlet type ventricular septal defect (24 mm) and very small secundum type atrial septal defect (3 mm) with left-to-right shunt was illustrated. Aorta was anterior and the left side of the pulmonary artery and originated from rudimentary RV. PA derived from the main ventricle with confluent pulmonary artery branches with mild pulmonary insufficiency and severe pulmonary hypertension (mPAP:70 mmHg). The thick and hypoplastic left A-V valve was remarkable with severe stenosis (MG:15 mmHg, PG:21 mmHg, Figures 1(a)–1(d)).

Based on the TTE, DILV with CCTGA (congenitally corrected transposition of great arteries) physiology and severe left A-V valve stenosis (DILV type AIII) was confirmed for better evaluation, and cardiac catheterization was performed.

**Figure 2:**
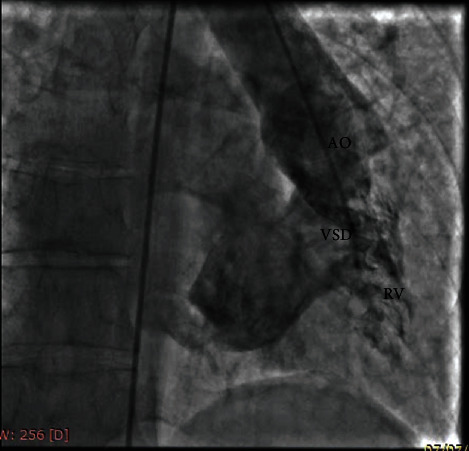
RV injection shows rudimentary systemic RV at the left side and large VSD.

Cardiac catheterization showed RV injection which demonstrated rudimentary RV at the left side; severe systemic ventricle (LV) enlargement with moderate dysfunction which filled via a large inlet type VSD. PA originated from LV (Figure 2). Both A-V valves were connected to LV with severe left A-V valve stenosis (MG: 15 mmHg).

Regarding to his refractory symptoms and significant left A-V valve stenosis and small ASD, he was scheduled for AFR implantation to reduce LA and pulmonary capillary wedge pressure.

### 2.1. Procedural Technique

**Figure 3:**
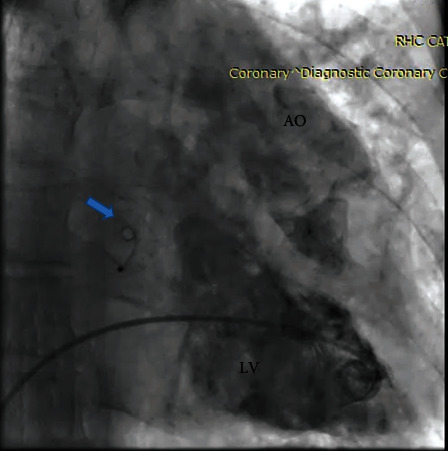
AFR is seen in the proper position.

The procedure was performed under local anesthesia and intensive hemodynamic monitoring. Through the right femoral vein, after heparin injection to achieve ACT > 250 s, under TEE guidance, the ASD was crossed with multipurpose catheter which its tip was positioned in the LUPV and a floppy tip Amplatzer super stiff guide wire placed in the LUPV; then, sequential balloon dilatation of the atrial septum was done with peripheral balloons 10-30 mm and 12-30 mm (OTW). After that, the delivery sheath (Occlutech 14F) was crossed through the wire to mid LA, and the dilator was removed to allow back bleed. After loading the device (AFR 10 mm) and connecting to the delivery cable, the device was passed over the sheath to reach the tip of the sheath. By retracting the sheath, the distal disk was deployed in LA; then, the whole system was pulled backed toward the septum; after checking the orientation of the device by TEE, the proximal disk deployed. Eventually, after complete assessment of the device position, its stability, and the degree of left to right shunt, the device was released (Figure 3). Immediately after device implantation, hemodynamic study showed reduction of wedge pressure to 18 mmHg. Hemodynamic study before and after intervention is shown in Table 1.

The patient's daily visit after the procedure until discharge (one week) indicated that his shortness of breath had decreased. Lower extremity edema decreased, and blood oxygen saturation reached 79%.

Furthermore, at 2 months follow-up, the patient's clinical status (dyspnea and edema) improved significantly; cyanosis was decreased, and So2 increased to 79%.

**Figure 4:**
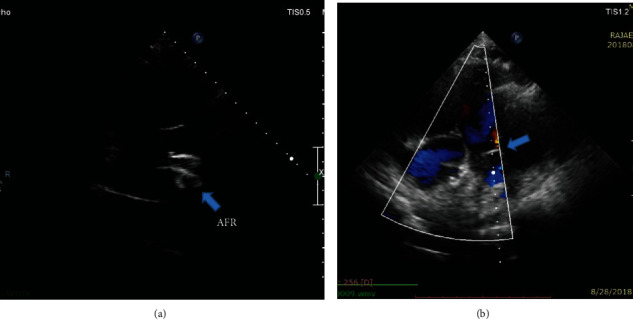
Follow-up echocardiography indicating the proper position of the AFR device with left-to-right shunt.

Follow-up echocardiography showed the AFR device in the proper position with left-to-right shunt, and the left A-V valve mean pressure gradient was decreased to 7 mmHg, and mPAP decreased from 80 to 50 mmHg (Figure 4).

## 3. Discussion

In this article, we review different anatomical and hemodynamic conditions in patients with DILV and review different treatment methods in these patients. In addition, we are reviewing a new palliative treatment that has been performed for the first time in Iran in our patient.

As will be discussed below, the mentioned patient was still symptomatic with full drug treatment, and due to his condition and progression of disease to the Eisenmenger, it was not possible to perform corrective surgery for him, and we had to undergo palliative treatment.

As mentioned in studies, the most prevalent form of the univentricular heart is double-inlet ventricle in which a large ventricle, mostly with left ventricle morphology, receives both atrioventricular valves and connects to a hypoplastic ventricle through a ventricular septal defect which is called bulboventricular foramen [5]. Based on study, we have some anatomic heterogeneity in this disease [6], and therefore, the clinical presentations are variable and for this reason, there are divergent methods for palliative therapy of these patients including modified Blalock-Taussig shunt (for those with restrictive pulmonary blood flow), pulmonary artery banding (for patients with unrestrictive pulmonary blood flow), modified Norwood operation, palliative arterial switch, or arch repair plus concomitant pulmonary artery banding (for patients with systemic outflow tract obstruction or arch obstruction) [7]. Based on mentioned points, however, recent advances in the treatment of this disease have led to a major breakthrough in early and late prognosis of these patients and for some, we may not be able to do so for a variety of reasons [8].

Furthermore, in patients with left A-V valve stenosis, there is pressure gradient between LA and LV which in long-term leads to pulmonary venous congestion, pulmonary artery hypertension, and finally, irreversible elevated pulmonary vascular resistance [9].

In our patient who did not undergo any palliative strategies in a timely manner, due to the severity of his symptoms and the outstanding elevation of capillary wedge pressure and no convenient surgical options, we decided to perform percutaneous therapeutic interventions.

We had two options: dilating the interatrial septum defect or commissurotomy of the left AV valve; because of unsuitable anatomy of the AV valve for balloon dilation, we chose the first option. Isolated balloon atrial septostomy (BAS) might lead to unintentional tearing of the IAS and potential death. Also, it might occlude within the short-term due to overgrowth of intimal cells. So, we arbitrated to deploy the AFR device at the IAS to ensure that the hole remained open permanently.

The AFR device is a self-expandable double-disc nitinol wire mesh production which creates a communication between the atriums and allows blood to flow across the interatrial septum [10]. Initially, it was designed for those with severe pulmonary hypertension and RV failure with refractory symptoms for decompressing the right heart chambers via permanent right-to-left shunt which leads to elevation of the cardiac output, blood pressure, and organ perfusion despite of inducing mild systemic desaturation. Following the more experience about the role of this device, nowadays, it could be used whenever the either atrial pressure increases; so, the device would decompress the dedicated atrium. Those with elevated left atrium pressure would create a persistent left-to-right shunt which cause the reduction of LAP, PCWP, PAP, and pulmonary congestion symptoms and increase arterial blood saturation, cardiac output, blood pressure, and organ perfusion [11].

The diameter of disc varied from 16 to 23, the fenestration diameter ranges between 4 and 10 mm. and disc connective waist depending on the thickness of atrial septum varied between 2 and 0 mm.

The device is very flexible and adaptable with distinctive braiding and is currently used in patients with severe right ventricular (RV) failure due to pulmonary hypertension or left heart failure [9], left atrial congestion, and failing Fontan [12].

## 4. Conclusion

Transcatheter AFR device implantation could be regarded as a relatively novel palliative approach that is utilized in selective high-risk patients with pulmonary hypertension (PH).

Although it was used to be implanted in pH patients with RV overload for decompressing the right side of the heart, in this case, we implanted that to decompress the left side chambers due to severe left A-V valve stenosis in DILV patient.

To our knowledge, this case is one of the first experiences with the Occlutech AFR device implementation in adult patient with cyanotic congenital heart disease, with severe AV valve stenosis and restricted atrial septal defect.

## Figures and Tables

**Table 1:** Hemodynamic parameters (mmHg) before and after AFR device implantation.

	LV	RV	PAP	Wedge	RA
Before	110/0-10	100/0-8	110/65	25	8
After	110/0-10	90/0-6	105/45	18	6

AFR: atrial flow regulator; LV: left ventricle; RV: right ventricle; PAP: pulmonary arterial pressure; RA: right atrium.

## Data Availability

The manuscript submitted here is a case report, and more details are available upon request to first author.
